# Supplementary material for: Management of childbearing with hypermobile Ehlers-Danlos syndrome and hypermobility spectrum disorders: A scoping review and expert co-creation of evidence-based clinical guidelines
Source: PLoS One. 2024 May 15;19(5):e0302401. doi: 10.1371/journal.pone.0302401 (PMC11095771; doi:10.1371/journal.pone.0302401)
Supplement: S2 File — Guidelines for physical therapy. (DOCX) [file pone.0302401.s002.docx]

# **S1. Supporting information. Physical therapy**

| **Physical Therapy** | |
| --- | --- |
| **Maintenance of overall wellbeing** | Consideration of one’s overall wellbeing, and quality of life is key. Best practice in this population is to use an individualized comprehensive integrative multi-modal approach to all health issues.   - Body mechanics, ergonomic and postural awareness may need to be modified with work activities, activities of daily living and childcare. - Pelvic girdle diagnostic tests need to be utilized with care and caution with hEDS/HSD. Test and assess with caution and to not provoke symptoms. - Diastasis recti abdominis should be screened post childbirth. - Individuals should seek care from a pelvic health rehabilitation for guidance and manual therapy as indicated.   **Physical wellbeing:**   - Those with hEDS/HSD access a rehabilitation health care professional such as a physical therapist or occupational therapist experienced with hEDS/HSD prior to pregnancy, during pregnancy and for a prolonged period postnatally (3-months) to reduce likelihood of chronic pelvic girdle pain. - Modified core strengthening exercises throughout pregnancy to maintain mobility and aid recovery. - Improve stability of the joints before and during pregnancy and optimize an often-compromised proprioception to mitigate the hormonal effects of loosened tissue during pregnancy.   **Exercise:**   - Follow existing guidance where feasible (30–60-minute sessions, 3-4 times per week (up to daily). - Session times should vary based on tolerance with sufficient rest time. - Moderate intensity (12–14 on Borg scale). - Supervision recommended. - All exercise during pregnancy should be modified and individualized for those with hEDS/HSD. - Adhere to existing guidance on typical warning signs to discontinue exercise during pregnancy. For those with hEDS/HSD these will also include increased pain and instability . - Monitor vital signs (high incidence of POTS). - Additional bracing and support may be indicated (e.g., back, abdominal and/or pelvic support with compression hosiery). - Exercises to improve performance of pelvic floor postnatally:   - back flexors.   - back extensors.   - hip extensors.   **Individualized therapy programs for the hEDS/HSD population should include:**   - individualized core stabilization. - joint stabilization. - proprioception and balance training as appropriate. - aquatics. - physio ball/exercise ball stability exercises. - closed chain activities - lumbar and abdominal strengthening. - mat exercise. - standing and sitting exercise. - functionally based activity training.   **Alternate bracing/support options:**   - Abdominal binders/maternal abdominal belly bands. - Pubic symphysis/sacro iliac joint belts. - Pelvic support belts (to address prolapse/vulvar edema). - Breast support (supportive breast support without placing stress on shoulders or back) - posture support bras.   **Recommended Modifications in Activities of Daily Living:**   - Seated posture (head/back support, foot support, depth of chair to fit body) - Standing posture (foot support/footwear, bracing as needed, compression socks/stockings, abdominal support). - Transitional movements (sit to stand, rolling, getting up and down from a lying position. Pillows may be helpful to squeeze between the knees during these movements). - Gait (abdominal bracing, footwear, canes, walkers, crutches, or scooters/ wheelchairs as indicated). - Task specific work activities (ergonomics, avoid prolonged positioning) - Movement modification with modified tools. - Self-care (e.g., bathing and foot care). - Sleep positions (use of pillows / props to support head, neck, torso, arms, and feet). |
